# Identifying Predictors of Psychological Distress During COVID-19: A Machine Learning Approach

**DOI:** 10.3389/fpsyg.2020.586202

**Published:** 2020-11-05

**Authors:** Tracy A. Prout, Sigal Zilcha-Mano, Katie Aafjes-van Doorn, Vera Békés, Isabelle Christman-Cohen, Kathryn Whistler, Thomas Kui, Mariagrazia Di Giuseppe

**Affiliations:** ^1^School-Clinical Child Psychology Program, Ferkauf Graduate School of Psychology, Yeshiva University, Bronx, NY, United States; ^2^Department of Psychology, University of Haifa, Haifa, Israel; ^3^Clinical Psychology Program, Ferkauf Graduate School of Psychology, Yeshiva University, Bronx, NY, United States; ^4^Department of Surgical, Medical and Molecular Pathology and of Critical Care Medicine, University of Pisa, Pisa, Italy

**Keywords:** COVID-19 pandemic, emotion regulation, somatization, machine learning, anxiety, depression, post-traumatic stress, defense mechanisms

## Abstract

Scientific understanding about the psychological impact of the COVID-19 global pandemic is in its nascent stage. Prior research suggests that demographic factors, such as gender and age, are associated with greater distress during a global health crisis. Less is known about how emotion regulation impacts levels of distress during a pandemic. The present study aimed to identify predictors of psychological distress during the COVID-19 pandemic. Participants (*N* = 2,787) provided demographics, history of adverse childhood experiences, current coping strategies (use of implicit and explicit emotion regulation), and current psychological distress. The overall prevalence of clinical levels of anxiety, depression, and post-traumatic stress was higher than the prevalence outside a pandemic and was higher than rates reported among healthcare workers and survivors of severe acute respiratory syndrome. Younger participants (<45 years), women, and non-binary individuals reported higher prevalence of symptoms across all measures of distress. A random forest machine learning algorithm was used to identify the strongest predictors of distress. Regression trees were developed to identify individuals at greater risk for anxiety, depression, and post-traumatic stress. Somatization and less reliance on adaptive defense mechanisms were associated with greater distress. These findings highlight the importance of assessing individuals’ physical experiences of psychological distress and emotion regulation strategies to help mental health providers tailor assessments and treatment during a global health crisis.

## Introduction

In March 2020, the World Health Organization declared the current outbreak of COVID-19, the disease caused by severe acute respiratory syndrome coronavirus 2 (SARS-CoV-2), a pandemic. At the time of this writing, there are more than 41 million confirmed cases of COVID-19 across 227 countries (World Health Organization, 2020) and the worldwide death toll has surpassed one million. Besides the obvious impact on physical health, the pandemic is likely to negatively affect mental health and well-being (Brooks et al., 2020; Qiu et al., 2020).

In tandem with living amidst a global pandemic, stress, social isolation, and the associated financial crisis, may result in significant adverse mental health effects. During the SARS epidemic in 2003, studies reported elevated levels of anxiety and depression that persisted 3 years later (Chan et al., 2006; Ko et al., 2006; Liu et al., 2012), with those under quarantine showing a dramatic increase in post-traumatic stress symptoms (PTSS; Hawryluck et al., 2004; Lau et al., 2005; Wu et al., 2005; Liu et al., 2012). Initial reports from China indicate that the COVID-19 outbreak and associated quarantining measures have also led to an increase in symptoms of psychological distress including anxiety, depression, self-harm, suicide attempts (Qiu et al., 2020), and post-traumatic stress (Liu et al., 2020). Similarly, during the acute phase of COVID-19 in Italy, the number of days in lockdown was associated with higher levels of psychological distress, including higher PTSS (Conversano et al., 2020a; Di Giuseppe et al., 2020c; Marazziti et al., 2020). A recent meta-analysis focused on prevalence rates for psychological distress during COVID-19, found high rates of anxiety (31.9%), depression (33.7%), and stress (29.6%) (Salari et al., 2020).

### Risk Factors for Psychological Distress

Specific populations are likely to be more vulnerable to the psychological impact of global health crises such as COVID-19 (for a review see Brooks et al., 2020). Among Chinese healthcare workers during COVID-19, women reported more severe symptoms of anxiety, depression, insomnia, and general distress and more severe PTSS and disrupted sleep than their male counterparts (Lai et al., 2020). Age also appears to be an important risk factor for psychological distress. While rates of mortality and illness severity are lower among young people infected with COVID-19, younger individuals have reported more adverse psychological consequences, such as anxiety, depression, and post-traumatic stress (Conversano et al., 2020b; Qiu et al., 2020).

Adverse childhood experiences (ACEs) also have a tremendous impact on general mental health and well-being across the lifespan (Felitti et al., 1998; Hughes et al., 2017). Although ACEs do not appear to have been researched in the context of pandemics, it is probable that individuals with childhood trauma are at increased risk for psychological distress during the COVID-19 crisis (Bryant et al., 2020). Other risk factors associated with adverse mental health outcomes include low socioeconomic status and being a racial and/or ethnic minority. The complex interaction of early childhood trauma, racial/ethnic identity, and socioeconomic status is a critical determinant of physical and mental health outcomes (Wilkinson and Marmot, 2003).

### Coping With Distress During COVID-19: Explicit and Implicit Emotion Regulation

Besides pre-existing risk factors, the way people cope with stressful events has also been shown to be important in mitigating psychological distress (Gross and John, 2003; Aldao et al., 2010). Individuals tend to utilize various explicit and implicit emotion regulation strategies to mitigate distress through modification of the intensity, duration, and type of the experienced emotion (Gross and Thompson, 2007; Gyurak et al., 2011). Whereas explicit emotion regulation requires voluntariness and effort, implicit emotion regulation is an ongoing, effortless, automatic process that operates outside of awareness. Although both are crucial in maintaining psychological well-being, there is evidence suggesting that implicit emotion regulation may be even more important to healthy psychological functioning than explicit emotion regulation (Gyurak et al., 2011). Specifically, in anxiety and depression, emotion dysregulation has been proposed to originate more in implicit, automatic processes rather than explicit ones (Ehring et al., 2010; Etkin et al., 2010).

### Explicit Emotion Regulation

Explicit emotion regulation is a conscious effort to control and change one’s initial emotional reaction. Two major strategies are cognitive reappraisal and expressive suppression (Gross and John, 2003). Reappraisal involves reinterpreting the meaning of an event to alter its emotional impact (Gross, 1998) and is generally considered to be an adaptive emotion regulation strategy associated with better interpersonal outcomes and well-being (Gross and John, 2003). In contrast, suppression involves an attempt to inhibit the expression of emotion and is associated with more psychological distress (Aldao et al., 2010).

### Implicit Emotion Regulation

#### Defense Mechanisms as Implicit Emotion Regulation

One strategy to implicitly regulate emotion is the use of defense mechanisms. Defense mechanisms fall on a continuum ranging from maladaptive defenses (e.g., acting out or passive aggression) to highly adaptive defenses (e.g., humor and altruism; Perry, 1990; American Psychiatric Association, 2000). Use of adaptive defenses can reduce the length or intensity level of distress, or can positively change the quality of an emotional response (Koole and Rothermund, 2011), while reliance on maladaptive defenses tends to contribute to the maintenance and exacerbation of psychopathology (Rice and Hoffman, 2014; Perry et al., 2020). Adaptive defense mechanisms are known to mediate more severe reactions to traumatic events (Riolli and Savicki, 2010). During the outbreak in Italy, individuals under quarantine who relied on maladaptive defenses had significantly higher levels of anxiety, depression, and PTSS as compared to people who used more adaptive defense mechanisms (Di Giuseppe et al., 2020a).

#### Somatization as Implicit Emotion Regulation

Another type of implicit emotion regulation strategy that might impact the level of psychological distress is somatization. Somatization refers to the presence of physical symptoms – such as pain, dizziness, and indigestion – that have no known organic cause (Greenberg, 2014). It is understood as a phenomenon in which difficult thoughts and emotions are expressed through medically unexplained physical symptoms (Chander et al., 2019; Fu et al., 2019). The presence of somatic symptoms is associated with difficulty experiencing, describing, and identifying emotions and a tendency to withdraw into fantasy (i.e., alexithymia; Bailey and Henry, 2007). Somatization thus blocks the experience of the original emotions, which may lead to greater distress (Katon et al., 2001; Abbass, 2005; Busch, 2014). Although there is overlap between somatic symptoms, anxiety, and depression (Löwe et al., 2008; Simms et al., 2012; Fu et al., 2019), somatization is a distinct phenomenon. Specifically, somatic symptoms (a) are associated with impairment in social functioning (Löwe et al., 2008); (b) result in greater healthcare utilization and medical care costs (Barsky et al., 2005); and (c) rely on different psychobiological pathways than related psychological disorders such as depression (Rief et al., 2010). Somatization has been implicated as a key factor in a range of anxiety and other disorders (Kroenke et al., 1994; Blaya et al., 2006) and has been identified as a distinct predictor of quality of life, independent of anxiety and depression (Hyphantis et al., 2010).

### The Current Study

All the factors described thus far impact mental health, but little is known about which variables have the most impact and how they interact with one another to predict psychological distress during a pandemic. Previous studies examined single risk factors (or a small set of risk factors) with statistical models that treat all other variables as merely noise. Testing each predictor factor as a separate hypothesis, as done in traditional statistical approaches, can lead to erroneous conclusions because of multiple comparisons (inflated type I errors), model misspecification, and multicollinearity. Unlike traditional statistical models, machine learning models are not constrained by assumptions and are particularly helpful for finding patterns in complex datasets (Orrù et al., 2020). Specifically, the random forest method is able to identify the most important predictors from a large set of potential predictor variables. Moreover, the subsequent regression tree analysis allows for the identification of various interactions between the predictor variables.

The aims of the current study were threefold: (1) To identify the prevalence of anxiety, depression, and posttraumatic stress among adults during COVID-19; (2) To determine the most prominent statistical predictors of anxiety, depression, and post-traumatic stress, using random forest machine learning models; (3) To explore how these predictors might interact in identifying individuals who are at a greater risk of psychological distress, using decision tree regression models.

## Methods and Materials

### Procedures and Participants

This cross-sectional study was advertised via social media and email listservs, with participants invited to complete an online Qualtrics survey. Data were collected between March 25, 2020 and April 22, 2020. At the conclusion of the survey, all participants were provided with links to resources supporting mental health and well-being during the pandemic.

The number of participants who provided consent was 3,192. Only those participants who proceeded beyond the demographic portion of the survey (*N* = 2,787) were included in this study. Detailed demographic data about the study sample is presented in Table 1.

**TABLE 1:** Demographic characteristics of the sample (*N* = 2,787).

Variable	*N* (%)
**Age**	
18–24	233 (8.3)
25–34	632 (22.7)
35–44	608 (21.8)
45–54	466 (16.7)
55–64	445 (16.0)
65–74	340 (12.2)
75–84	56 (2.0)
85+	7 (0.3)
**Gender**	
Female	2,268(81.4)
Male	470 (16.9)
Non-binary	48 (1.7)
**Race/ethnicity**	
White	2,232(80.1)
Asian or Asian Indian	271 (9.7)
Hispanic/Latino	125 (4.2)
Black	63 (2.2)
Middle Eastern/North African	37 (1.3)
Native American/Aboriginal	26 (0.9)
Multiracial/multi-ethnic	16 (0.6)
Pacific Islander	11 (0.4)
**Country**	
United States	1,931(69.2)
Australia	579 (20.8)
China	109 (3.9)
United Kingdom	31 (1.1)
Canada	20 (0.7)
Netherlands	20 (0.7)
Hungary	17 (0.6)
All other countries	66 (2.3)
Missing	14(<0.5)
**Education**	
<High school	47 (1.7)
High school graduate	201 (7.2)
Some college	477 (17.1)
2-year degree	200 (7.2)
4-year degree	739 (26.5)
Professional degree	784 (28.1)
Doctorate	338 (12.1)
**Socioeconomic class**	
Lower class	247 (7.7)
Lower middle class	631 (19.8)
Middle class	1,374(43.0)
Upper middle class	258 (23.7)
Upper class	104 (3.4)
**Marital status**	
Married	1,502(53.9)
Single/never married	803 (28.8)
Divorced/separated	399 (14.3)
Widowed	82 (2.9)
**Know someone diagnosed with COVID-19**	
Yes	927 (33.3)
No	1,860(66.7)
**Know someone who has died of COVID-19**	
Yes	199 (7.1)
No	2,588(92.9)

### Measures

#### Predictors

##### Demographics

Participants provided information for the following candidate predictors: age, gender, country of residence, ethnicity, socio-economic status, education level, marital/relationship status. In addition, participants reported whether they had a pre-existing chronic health condition, knew someone diagnosed with coronavirus, knew someone who had died as a result of COVID-19, were a frontline healthcare worker, and/or work in another industry deemed essential (e.g., cashiers, delivery services).

##### Childhood trauma

Participants completed the 10-item Adverse Childhood Experiences Questionnaire (Felitti et al., 1998). This measure asks about individuals’ experience of abuse, neglect, and household dysfunction prior to the age of 18. The test-retest reliability ranges from 0.52 to 0.72 (Dube et al., 2004). The test-retest reliability for emotional abuse, physical abuse, and sexual abuse is 0.66, 0.55, and 0.69, respectively (Dube et al., 2004). In the present study, internal consistency for the ACE was 0.77.

##### Explicit emotion regulation

Key aspects of explicit emotion regulation were assessed with the Emotion Regulation Questionnaire (ERQ; Gross and John, 2003). The ERQ includes 10 items that measure respondents’ tendency to regulate their emotions through cognitive reappraisal and expressive suppression. Respondents answer each item on a 7-point Likert-type scale ranging from 1 (strongly disagree) to 7 (strongly agree). The ERQ has been used extensively in research on emotion regulation and demonstrates acceptable internal consistency and construct validity (Gross and John, 2003). The two-factor model is replicable in community samples and internal consistency for the subscales is acceptable to excellent, cognitive reappraisal (α = 0.89–0.90) and expressive suppression (α = 0.76–0.80; Preece et al., 2020). Internal consistency in the current study was 0.86 for the cognitive reappraisal subscale and 0.79 for the expressive suppression subscale.

##### Implicit emotion regulation

Defense mechanisms were assessed with the Defense Mechanisms Rating Scale – Self Report (DMRS-SR-30; Di Giuseppe et al., 2020b) a 30-item inventory that assesses defense mechanisms across the hierarchy described in the Diagnostic and Statistical Manual of Mental Disorders, 4th Edition (DSM-IV; American Psychiatric Association, 2000). The DMRS-SR-30 uses a 5-point Likert scale ranging from 0 (not at all) to 4 (very often/much). The questionnaire assesses 28 defenses and provides proportional scores for seven hierarchically ordered defense levels. The levels, ranging from most to least adaptive, are: adaptive/mature, obsessional, neurotic, minor image-distortion/narcissistic, disavowal, major image-distortion/borderline, and action. The psychometric properties of this DMRS-SR-30 are robust, with internal consistency ranging from good to excellent across all subscales and strong convergent and divergent validity (Di Giuseppe et al., 2020b).

Somatization was measured with the PHQ-15 (Kroenke et al., 2002) which asks about 15 somatic symptoms that account for 90% of the symptoms reported in outpatient settings (Kroenke, 2003). Items such as stomach pain, dizziness, and constipation are rated from 0 (not bothered at all) to 2 (bothered a lot). Total PHQ-15 scores range from 0 to 30 with scores of 0–4, ≥5, ≥10, and ≥15 representing minimal, mild, moderate, and severe levels of somatization, respectively (Kroenke, 2003). Internal consistency of α = 0.80–0.87 and test-retest reliability of 0.65 has been reported (Gierk et al., 2015). Internal consistency for the PHQ-15 in the current study was 0.78.

#### Outcome Variables

##### Psychological distress

Depression and anxiety were assessed with subscales of the Patient Health Questionnaire (PHQ; Spitzer et al., 1999), a screening tool for mental health disorders that is quick and easy for participants to complete. The PHQ includes the Patient Health Questionnaire for Depression (PHQ-9; Kroenke et al., 2001) and the Generalized Anxiety Disorder Scale (GAD-7; Spitzer et al., 2006). The cutoff score of 10 on the PHQ-9 has a sensitivity and specificity of 88% for major depression and was used in this study (Manea et al., 2012). For the PHQ-9, scores of 5–9, 10–14, 15–19, and 20–27 corresponded to mild, moderate, moderately severe, and severe depression symptoms, respectively.

For anxiety, this study used the GAD-7 module in the full PHQ. Participants completed this module only if they endorsed being bothered in the past 4 weeks by “feeling nervous, anxious, on edge, or worrying about a lot of different things.” This module of the GAD-7 asks participants to rate the presence of symptoms on a 3-point scale ranging from not at all (0) to more than half the days (2) during the past 4 weeks. Items are summed to create a severity score ranging from 0 to 14. Participants were considered to meet the criteria for GAD if the total score was ≥8 and three or more of the items were rated “more than half the days” (Terrill et al., 2015).

Both self-administered rating scales are based on the DSM-IV criteria for major depression and GAD. The PHQ and its modules for the various diagnostic categories have been used extensively and its reliability and validity are well-documented in the literature (Spitzer et al., 1999; Kroenke et al., 2010). Internal consistency for the PHQ-9 and the GAD-7 module in the current study was 0.91 and 0.81, respectively.

Post-traumatic stress symptoms were assessed with the Impact of Event Scale – Revised (IES-R; Weiss and Marmar, 2004), a 22-item self-report measure that assesses subjective distress caused by traumatic events. Following protocols used in numerous studies during pandemics, participants were asked to respond to the items with reference to the COVID-19 pandemic as the identified stressor. The IES-R yields a total score (ranging from 0 to 88). The recommended cutoff score of 33, suggesting a probable diagnosis of PTSD, was used in the current study (Creamer et al., 2003). In the current study, Cronbach’s alpha for the IES-R total score was 0.94.

### Data Analysis

The primary aim of the analysis was to develop a model to statistically predict the level of psychological impact of COVID-19. Following an initial examination of the data, the data was randomly separated into two parts; a training set of 70% of the total sample and a testing set of the other 30% of the dataset. For cross-validation, a machine learning model was first developed in the training set and subsequently tested in the separate testing set. In the present study, we sought to integrate the benefits of the predictability and interpretability of models (Shmueli, 2010; Yarkoni and Westfall, 2017), by (a) identifying the most predictive risk factors using machine learning models, and (b) providing interpretation by exploring how the risk factors interact in predicting symptom severity using traditional regression models.

#### Identifying Predictors of Symptom Severity

To identify the strongest predictors of symptom severity for anxiety, depression, and PTSS, a random forest algorithm (as implemented in the R package Random Forest version 4.6) was used. In this method, 500 regression trees were constructed based on bootstrapped samples from the primary dataset. For each tree, the recursive partitioning searches for binary splits in the sample that result in the smallest within-node sum of squared residuals. The procedure uses a random sample of partitioning variables for splitting at each node (i.e., potential split-point). In each leaf (i.e., split) of the tree, we estimated symptom severity. Final model predictions were obtained by aggregating the predictions across the trees. Cross-validation was used to reduce the number of splits in the tree (i.e., to set the minimum leaf size for splitting). To impute missing observations in the predictors, we used the R package missForest. For cross-validation, the models were fit on the training set and tested on the remaining 30% in the test set. Random forests were built for each psychological distress measure separately.

#### Estimating the Importance of Potential Predictors

To identify the strength of potential predictors, we used random forest to obtain a variable-importance plot, using conditional permutation (Strobl et al., 2008), that reflects the contribution of each variable to predicting symptom severity (Breiman, 2001). This method is a way of estimating each variable’s contribution to the prediction of outcome variables. We calculated an importance statistic reflecting the importance of each variable in producing accurate predictions for the outcome variables of anxiety, depression, and PTSS.

#### Exploring Interactions Between Potential Risk Factors in Predicting Outcomes

To complement the random forest analysis, we conducted a separate regression tree analysis focused on exploring how potential risk factors may interact to predict symptom severity. Regression trees were produced using Recursive Partitioning (RPART) analysis. All potential risk factors were entered into a regression tree analysis with the R package “rpart” (Breiman et al., 1984). The final tree was obtained by limiting the node size and pruning it by limiting its complexity according to cross-validation estimated prediction error.

## Results

From the 2,787 participants who proceeded beyond the demographic portion of the survey, the overall prevalence of acute levels of anxiety, depression, and PTSS was 27.3, 36.6, and 30.9%, respectively (see Table 2). These rates exceed past-year and lifetime prevalence for generalized anxiety (2.7 and 5.7%, respectively), depression, (6.8 and 16.9%), and post-traumatic stress disorder (3.6 and 6.8%; Kessler et al., 2004). Rates of distress in the current study also exceed those reported amidst the SARS pandemic. For example, during the SARS outbreak the prevalence of anxiety, depression, and PTSS was 13 (Wu et al., 2005), 8.8 (Liu et al., 2012) to 18 (Wu et al., 2005), and 4% (Wu et al., 2005), respectively.

**TABLE 2:** Prevalence of symptoms of psychological distress above the clinical cutoff.

	*N* (%)
No significant symptoms	1458 (52.3)
Anxiety symptoms only	129 (4.6)
Depression symptoms only	195 (7.0)
PTSS only	120 (4.3)
Anxiety + depression	205 (7.4)
Anxiety + PTSS	84 (3.0)
Depression + PTSS	71 (2.5)
Anxiety + depression + PTSS	525 (18.8)

*PTSS, post-traumatic stress symptoms. The following cutoff scores were used to classify those who were symptomatic. For depression, a cutoff score of 10 on the PHQ-9, representing moderate levels of depression, was used in this study (Manea et al., 2012). For generalized anxiety, participants with scores ≥8 on the GAD-7 module, who also had three or more items rated as “more than half the days” were considered to be symptomatic (Terrill et al., 2015). The recommended cutoff score of 33 on the IES-R, suggesting a probable diagnosis of PTSD, was used (Creamer et al., 2003).*

Some participants in the current sample experienced either symptoms of anxiety, depression, or post-traumatic stress, however, many (*N* = 885) experienced a combination of different symptoms. See Table 2 for an overview of reported symptom levels and comorbidities.

Prevalence of distress differed across demographic groups, in that women, non-binary participants, and younger participants (<45 years) reported significantly higher prevalence of all symptoms across all measures of distress. There was a statistically significant difference between all three gender groups for each symptom category as determined by one-way ANOVA (*p* < 0.001 for all comparisons). The largest effect sizes, though small, were found in the comparison of male to non-binary participants (Table 3).

**TABLE 3:** Symptoms of anxiety, depression, and post-traumatic stress by gender.

Symptom category	*M* (SD)	Effect size (Cohen’s *d*)
**Anxiety (GAD-7)**		
Male (*N* = 260)	8.35 (6.66)	Male vs. female = −0.09
Female (*N* = 1765)	7.48 (3.35)	Male vs. non-binary = −0.28
Non-binary (*N* = 38)	9.87 (2.98)	Female vs. non-binary = −.19
**Depression (PHQ-9)**		
Male (*N* = 454)	6.54 (6.86)	Male vs. female = −0.08
Female (*N* = 2,221)	8.62 (6.51)	Male vs. non-binary = −0.23
Non-binary (*N* = 47)	13.15 (7.62)	Female vs. non-binary = −0.16
**PTSS (IES-R)**		
Male (*N* = 429)	17.54 (16.17)	Male vs. female = −0.14
Female (*N* = 2112)	26.47 (16.80)	Male vs. non-binary = −0.26
Non-binary (*N* = 45)	35.80 (19.21)	Female vs. non-binary = −0.13

*Differences between all groups are significant at the p < 0.001 level. Questions about anxiety were only presented to participants who endorsed feeling nervous or anxious in the past 4 weeks.*

To evaluate age differences, participants were categorized into two age groups with younger <45 years and older ≥45 years. There was a statistically significant difference between groups for anxiety [*t*(2,061) = 2.62, *p* = 0.009, Cohen’s *d* = 0.05], depression [*t*(2,720) = 7.47, *p* < 0.001, *d* = 0.27], and PTSS [*t*(2,584) = 7.29, *p* < 0.001, *d* = 0.23].

Variables for race and ethnicity were transformed into a binary of White and all others (including those who endorsed the following racial and ethnic identities: Hispanic/Latino/Spanish, Black, Asian, Native American/Aboriginal, Middle Eastern/North African, Pacific Islander, and multiracial). Contrary to expectations, there were no differences between White participants and participants of color on symptoms of anxiety [*t*(2,061) = −0.31, *p* = 0.76], depression, [*t*(2,720) = 0.30, *p* = 0.76], or PTSS [*t*(2,584) = 1.34, *p* = 0.18].

To examine the relationship between self-perceived socioeconomic class (“How would you describe your socioeconomic status?”) and distress, socioeconomic class was transformed into a categorical variable with three levels. Group 1 consisted of “lower class” and “lower middle class” combined; Group 2 included “middle class” as its own category; and Group 3 was “upper middle class” and “upper class” combined. There were significant differences between the three groups for anxiety [*F*(2, 2,060) = 31.73, *p* < 0.001], depression, [*F*(2, 2,719) = 66.60, *p* < 0.001], and PTSS [*F*(2, 2,583) = 14.86, *p* < 0.001]. *Post hoc* comparisons were conducted using Tukey’s HSD (see Table 4) and indicated that individuals who described themselves as “lower class” and “lower middle class” reported higher levels of distress, particularly in comparison to individuals who described themselves as “upper middle class” or “upper class”; however, the effect sizes for these differences were relatively small.

**TABLE 4:** *Post hoc* comparisons for anxiety, depression, and post-traumatic stress by socioeconomic class.

	Group 1	Group 1	Group 1	Summary
	*M* (*SD*)	*M* (*SD*)	*M* (*SD*)	
Anxiety	8.25 (3.45) (*N* = 601)	7.17 (3.35) (*N* = 862)	6.77 (3.26) (*N* = 600)	G1 > G2 (*d* = 0.08) G1 > G3 (*d* = 0.11) G2 = G3
Depression	10.57 (7.31) (*N* = 754)	7.92 (6.46) (*N* = 1,197)	6.86 (5.68) (*N* = 771)	G1 > G2 (*d* = 0.10) G1 > G3 (*d* = 0.14) G2 > G3 (*d* = 0.04)
Post-traumatic stress	28.01 (18.04) (*N* = 725)	23.77 (16.87) (*N* = 1,130)	24.36 (15.91) (*N* = 731)	G1 > G2 (*d* = 0.06) G1 > G3 (*d* = 0.05) G2 = G3

*Group 1, “lower class” and “lower middle class”; Group 2, “middle class”; Group 3, “upper middle class” and “upper class.” Post hoc comparisons conducted with Tukey’s HSD. Results are summarized in the last column (p < 0.001).*

Among participants who completed all questions on the ACE (*N* = 2,157), 21% endorsed four or more ACEs. As expected, higher numbers of ACEs were associated with higher self-reported anxiety [*r*(1,684) = 0.28, *p* < 0.001], depression [*r*(2,140) = 0.32, *p* < 0.001], and PTSS [*r*(2,155) = 0.27, *p* < 0.001] symptoms.

### Predicting Risk Factors for Anxiety, Depression, and Post-traumatic Stress

Among participants (*N* = 2,787) who proceeded beyond the demographic portion of the survey, responses from 551 individuals were removed because they had more than 10% missing data in the remainder of the survey. Most of these removed participants discontinued participation before completing measures of implicit and explicit emotion regulation. The subsequent results are based on responses from the remaining 2,236 participants.

#### Anxiety

**FIGURE 1:**
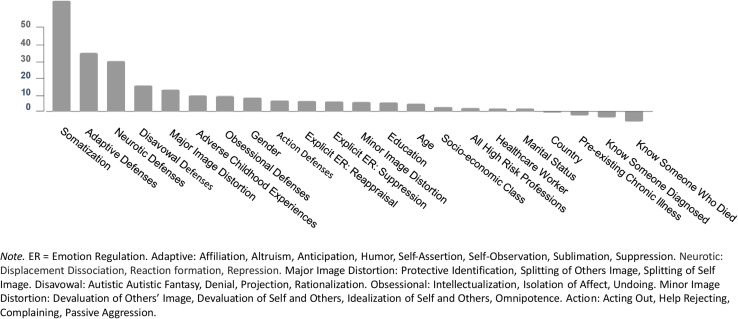
Predictors of anxiety.

The predictors of anxiety, according to their order of importance, appear in Figure 1. We used the resultant random forest to predict anxiety in the testing set. The correlation between predicted values in the training set and observed values in the test set was 0.90. Graphs for predicted vs. observed anxiety appear in the online supplements (Supplementary Figure 1).

**FIGURE 2:**
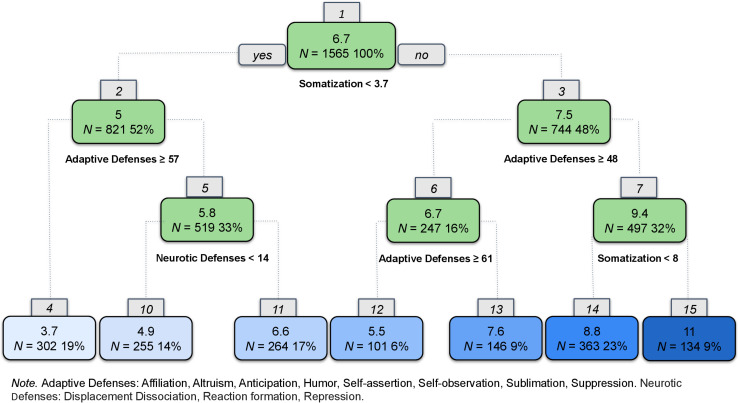
Regression tree for anxiety.

In the second step, we tested the ability to fit a single regression tree for anxiety (Figure 2). High somatization and less reliance on adaptive defenses predicted higher anxiety. High somatization was indicated in Node 1 with scores on the PHQ-15 ≥ 3.7; a second split at Node 7, with scores ≥8, predicted the highest levels of anxiety. Splits for adaptive defenses (*M* = 48.63, SD = 16.45, range = 0–100), such as humor, altruism, and affiliation, appear at Nodes 2, 3, and 6. There was also a split at Node 5 indicating that greater use of neurotic defenses, such as displacement, dissociation, reaction formation, and repression, were predictive of slightly more anxiety. Conversely, less somatization, more adaptive defenses, and fewer neurotic defenses appeared to predict lower levels of anxiety symptoms.

For this step, the correlation between predicted values of the training set and the observed values in the test set was 0.67 (Supplementary Figure 1). This metric provides an unbiased measure for the prediction accuracy of the model. Finally, we conducted a linear regression on the training set, focused on how potential risk factors may interact to predict symptom severity. The correlation between the predicted values and the actual values was 0.71.

#### Depression

**FIGURE 3:**
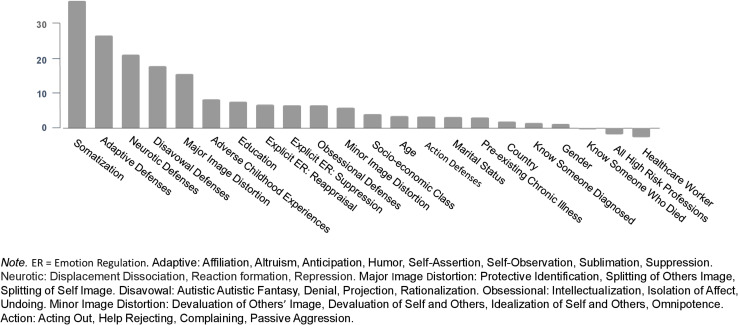
Predictors of depression.

The predictors of depression, according to their order of importance, appear in Figure 3. The correlation between the predicted values in the training set and observed values in the test set was 0.66 (see graph in Supplementary Figure 2).

**FIGURE 4:**
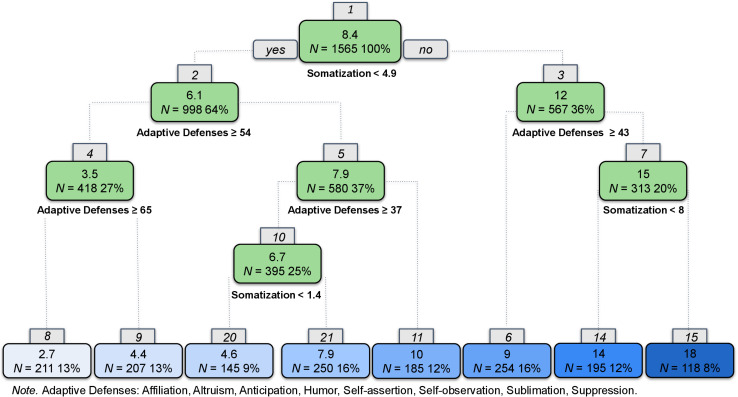
Regression tree for depression.

We followed the same data analytic strategy outlined for anxiety. The resulting regression tree for depression appears in Figure 4. High somatization and less reliance on adaptive defenses predicted the highest levels of depression, whereas low somatization and high reliance on adaptive defenses predicted lower levels of depression symptoms. High somatization was indicated at Node 1 with scores ≥4.9 and again at Node 7 (≥8). Node 10 shows that slightly elevated somatization makes another split among a subgroup of people who tend not to somatize (Node 1) and have moderate levels of adaptive defenses (Nodes 2 and 5).

The correlation between predicted values on the validation set and the observed was 0.67. The figures of observed vs. predicted of both models appear in the online supplements (Supplementary Figure 2). Finally, we examined a linear regression with all variables in the model and obtained a correlation of 0.72.

#### Post-traumatic Stress

**FIGURE 5:**
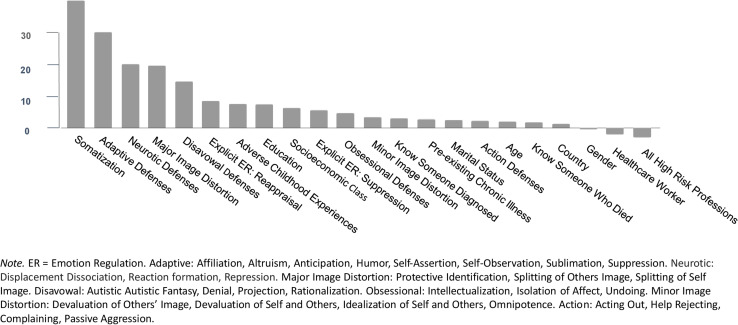
Predictors of post-traumatic stress symptoms.

Predictors of post-traumatic stress, according to their order of importance, appear in Figure 5. The correlation between the predicted and observed was 0.74. The graphs for predicted vs. observed appear in the Supplementary Figure 3.

**FIGURE 6:**
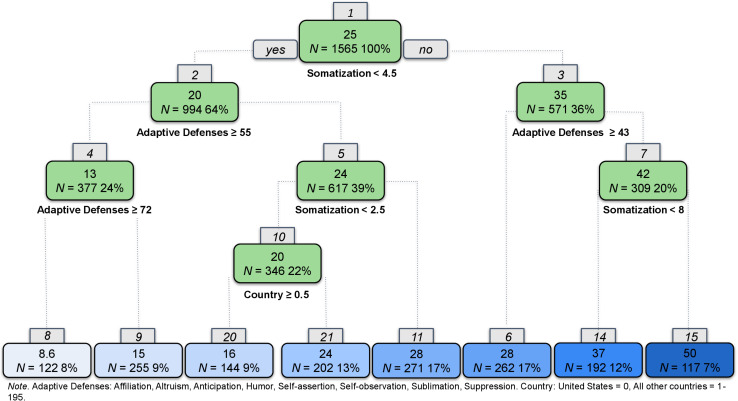
Regression tree for post-traumatic stress symptoms.

Following the same approach as was used for anxiety and depression, we developed a classification and regression tree for PTSS (see Figure 6). High levels of somatization and low levels of adaptive defenses predicted the highest level of PTSS. However, unlike the findings for anxiety and depression, a split at node 10 indicated that respondents from the United States reported significantly higher levels of PTSS compared with their global counterparts.

The correlation between predicted values on the training set and observed values in the test set was 0.63. The figures of observed vs. predicted models appear in the Supplementary Figure 3. Finally, we examined a linear regression with all variables in the model and obtained a correlation of 0.69.

## Discussion

This study examined the prevalence of psychological distress experienced during the COVID-19 pandemic and aimed to identify the strongest statistical predictors of distress. We found high levels of psychological distress exceeding prevalence rates in the general population absent a pandemic (Kessler et al., 2004) and rates of distress during previous global pandemics (Wu et al., 2005; Liu et al., 2012). These findings are in concert with other recent studies that have reported similarly high prevalence rates for anxiety, depression, and PTSS during the COVID-19 pandemic (Salari et al., 2020). The machine learning model for the prediction of anxiety, depression, and PTSS suggests that individuals who struggle to deal with pandemic-related stressors in adaptive ways, by relying more on somatization and less on adaptive defenses, may be more vulnerable to developing psychiatric symptoms (Perry et al., 2020).

Participants who were female and/or younger were more likely to report psychological distress. Although only a small sub-sample of this study, participants with a non-binary gender also appeared to be more vulnerable to experience psychological distress. This finding is supported by the existing literature on increased mental health risks for non-binary and genderqueer individuals (Budge et al., 2014; Matsuno and Budge, 2017) and suggests the continued importance of gender-affirming mental healthcare during a pandemic (American Psychological Association, 2015). There were no differences between White participants and participants of color in terms of anxiety, depression, and PTSS. This may be due to variability within different racial and ethnic groups in terms of economic stability, exposure to the virus, and other related factors (Himle et al., 2009; Novacek et al., 2020). There were, however, differences across all symptom categories when socioeconomic groups, albeit with small effects. This may point to the role of economic stratification and its impact on stressors such as unemployment and working conditions in low-wage jobs during the pandemic (Kantamneni, 2020).

Among the predictor variables, two forms of implicit emotion regulation – somatization and defense mechanisms – emerged as the most impactful factors in statistically predicting symptoms of anxiety, depression, and post-traumatic stress in our regression models. The results regarding somatization are in line with previous research findings about elevated somatic symptoms among traumatized individuals (Rohlof et al., 2014). Illness anxiety is naturally elevated during a pandemic. This increased emotional burden can translate into somatic symptoms in more vulnerable individuals, which, in turn, may lead to more psychological distress (Hyphantis et al., 2010). Although the cross-sectional nature of our study design prevents us from making causal inferences, the relationship between somatization and post-traumatic stress and anxiety could be understood in both directions: somatization as a vulnerability factor may lead to more anxiety, depression, and PTSS, and vice versa, experiencing psychological distress in the form of anxiety, depression, and PTSS may make individuals prone to develop somatization symptoms. In addition, it is possible that somatization and psychological distress negatively impact each other: the distress can easily translate into somatic symptoms in more vulnerable individuals, which, in turn, may lead to more psychological distress.

Participants in the United States (who also met the splits at Nodes 1, 2, and 5) had significantly higher levels of PTSS compared to their global counterparts, with American participants more likely to cross the threshold for likely PTSD (score of 24 on the IES-R). This suggests that individuals outside of the United States, with relatively healthy implicit emotion regulation strategies, were less likely to experience distress; whereas American participants with similar implicit emotion regulation strategies were more likely to experience a clinical level of PTSS. This difference may be due to poor access and affordability of healthcare in the United States (Ginsburg et al., 2008; Schoen et al., 2013). It is also possible that people in the United States were exposed to more traumatic experiences during the time of data collection, from mid-March to mid-April 2020, compared to participants in other countries.

In contrast with expectations based on previous trauma literature (Burns et al., 2010; Powers et al., 2015; Westermair et al., 2018), ACEs (though associated with symptoms of anxiety, depression, and PTSS) were not identified as a predictor of distress. The fact that implicit emotion regulation processes were more predictive of psychological distress during the COVID-19 pandemic than ACEs, is a promising finding. It may indicate that vulnerability factors may be reduced, since, although childhood trauma cannot be undone, new more adaptive emotional regulation strategies can be learned.

Notably, explicit emotion regulation strategies did not appear to statistically predict psychological wellbeing during the pandemic. This highlights the salience of implicit ways of coping and suggests the importance of interventions that focus on identifying and modifying these capacities (Heldt et al., 2007; Babl et al., 2019; Kramer et al., 2010; Perry et al., 2020).

### Strengths and Limitations

This study extends beyond previous studies that identified several risk factors of psychological distress, by examining the interacting effects of these risk factors. A combined model that focused on prediction (random forest based on 500 trees) and explanation (regression single tree analyses) was used. Random forest analysis was used to identify the strongest statistical predictors and decision tree regression models helped explain how these predictors interact and impact anxiety, depression, and PTSS. This study also highlights the importance of specific implicit emotion regulation strategies.

There are several limitations worth considering. First, the cross-sectional design did not allow for empirically establishing causal relationships between predictor and outcome variables. Moreover, the use of online volunteers introduces both benefits and limitations. Research conducted online often results in more diverse samples that can be obtained rapidly, at lower cost, and with valid results (Casler et al., 2013; Shapiro et al., 2013; Chandler and Shapiro, 2016). However, online respondents may respond in an inattentive or non-serious manner (Aust et al., 2013; McKay et al., 2018). Although this study utilized commonly recommended tools for increasing validity of online research, including checks for unique IP addresses, completion time, and implausible answer combinations (Aust et al., 2013), it did not include specific validation questions or explicit questions about the seriousness of respondents (McKay et al., 2018). Additionally, there may have been a selection bias in that those who chose to respond to this study may have been experiencing greater distress during the pandemic than the population at large.

The use of brief screening measures provides only initial information about whether psychological distress has surpassed a threshold for acuity. Although the measures used in this study have well-documented predictive validity for DSM-5 diagnoses, they are not comprehensive diagnostic assessments. Additionally, the use of self-report measures for implicit emotion regulation presents an inherent challenge; there are remaining questions about the validity of self-report for implicit processes (Hofmann et al., 2005).

Although the use of machine learning in this study is innovative, several potential limitations must be highlighted. First, although psychologists might deem the sample large, and decision tree models have been applied to similarly sized datasets in the field of psychology (e.g., Delgadillo and Salas Duhne, 2020), for computer scientists this was a modest dataset. The required minimum sample size in machine learning is a fertile ground of methodological discussion. The ideal sample size needed for machine learning depends on the quality of data and the complexity of the model; however, the general rule of thumb is that the amount of training data needed for a well performing model is 10 times the number of parameters in the model (Caballero et al., 2006).

The present study reports on a rigorous cross-validation method for producing results that is likely to be generalizable to the broader population. However, there is a risk of identifying predictors in the test and validation samples that may not be as important in a new sample (Aafjes-van Doorn et al., 2020). Although the absence of out-of-sample external validation is common in mental health machine learning research (Aafjes-van Doorn et al., 2020), an additional step of out-of-sample validation would certainly strengthen the external validity of the findings (Sammut and Webb, 2017).

Perhaps the most significant limitation of psychological research during a pandemic is the inability to identify precipitating causes of distress. While the high rates of distress identified in the current study stand out, absent an available comparison sample (i.e., one unaffected by the pandemic) we cannot be certain that these increases and the identified predictor variables are completely unique to the pandemic. Anxiety, depression and PTSS are multiply determined. Amidst a global health crisis that has resulted in a radical shift in our way of life, rampant unemployment, and extraordinary physical distancing measures, it is difficult to determine whether distress is due to the pandemic itself, concomitant measures to contain the virus, social isolation and lack of social support (which the current study did not assess), economic burden, or some combination of these and other factors. We suspect it is the latter and that it would be difficult, if not impossible, to disentangle these variables.

## Implications and Conclusion

The COVID-19 pandemic is still unfolding, and it is likely that the virus and its consequences will impact the global population for some time to come. This study begins to answer the call to monitor rates of depression, anxiety and PTSS and to identify mechanisms that can help explain differential trajectories of distress during the COVID-19 pandemic (Holmes et al., 2020). The current findings have implications for primary care and mental health providers, many of whom are providing care online (van Daele et al., 2020). Healthcare providers may need to be vigilant for evidence of somatization and difficulties defending against distress when assessing patients who present for care, whether for COVID-related symptoms or unrelated difficulties during the COVID-19 pandemic. Implicit emotion regulation can be assessed with the same, freely available measures used in this study and confirmed with a medical assessment of potential causes of physiological symptoms.

The findings in the current study dovetail with other COVID-19 research on psychological distress amidst the pandemic (Mazza et al., 2020; Qiu et al., 2020) and highlight the public mental health crisis that is unfolding. There will undoubtedly be increased demand for mental health services in the coming years. It is essential that primary care and mental healthcare providers be equipped to respond to this dire need (Pfefferbaum and North, 2020). Assessing patients for somatization and ability to cope with ongoing stressors, should be a central part of any evaluation. The increase in telepsychotherapy may afford patients greater access to high-quality mental healthcare that can improve mental health outcomes and support resilience during the COVID-19 pandemic.

## Data Availability Statement

The datasets presented in this article are not readily available because the dataset is not approved by the IRB for use by other researchers. Requests to access the datasets should be directed to tracy.prout@yu.edu.

## Ethics Statement

The studies involving human participants were reviewed and approved by the Western Institutional Review Board for Yeshiva University. Participants provided written informed consent to participate in this study.

## Author Contributions

TAP led the development, conceptualization, and execution of the research. SZ-M analyzed and interpreted the data and assisted in writing. KA and VB contributed to the methodology, participant recruitment, data analysis, and writing. IC-C, KW, and TK assisted in reviewing the literature and writing the manuscript. MD assisted in methodology, scoring of measures, and writing the manuscript. All authors contributed to the article and approved the submitted version.

## Conflict of Interest

The authors declare that the research was conducted in the absence of any commercial or financial relationships that could be construed as a potential conflict of interest.
