# Supplementary figures and images for: Identifying Predictors of Psychological Distress During COVID-19: A Machine Learning Approach
Source: Front Psychol. 2020 Nov 5;11:586202. doi: 10.3389/fpsyg.2020.586202 (PMC7682196; doi:10.3389/fpsyg.2020.586202)

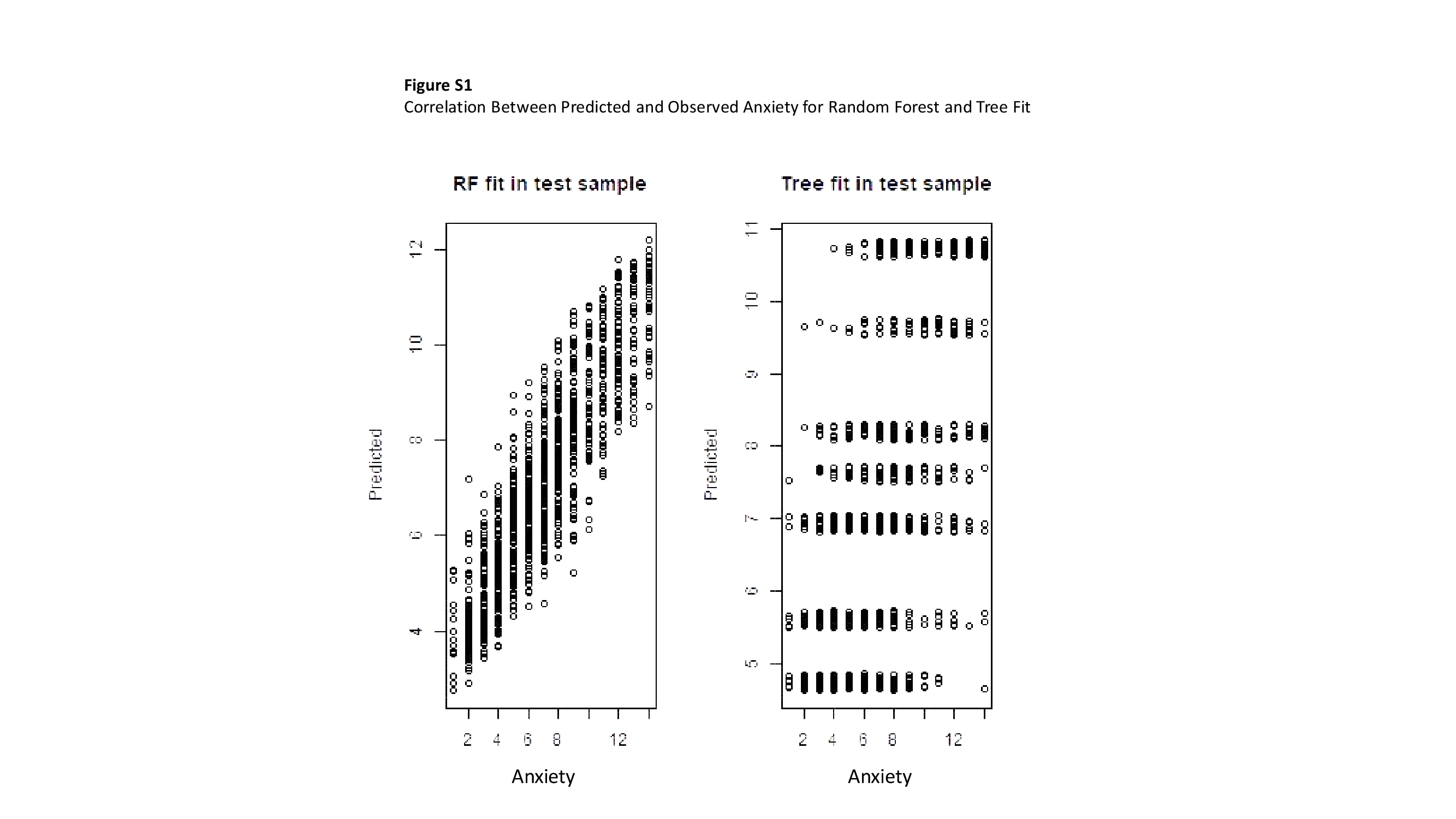

Supplement: Supplementary file 1 [file Image_1.TIFF]

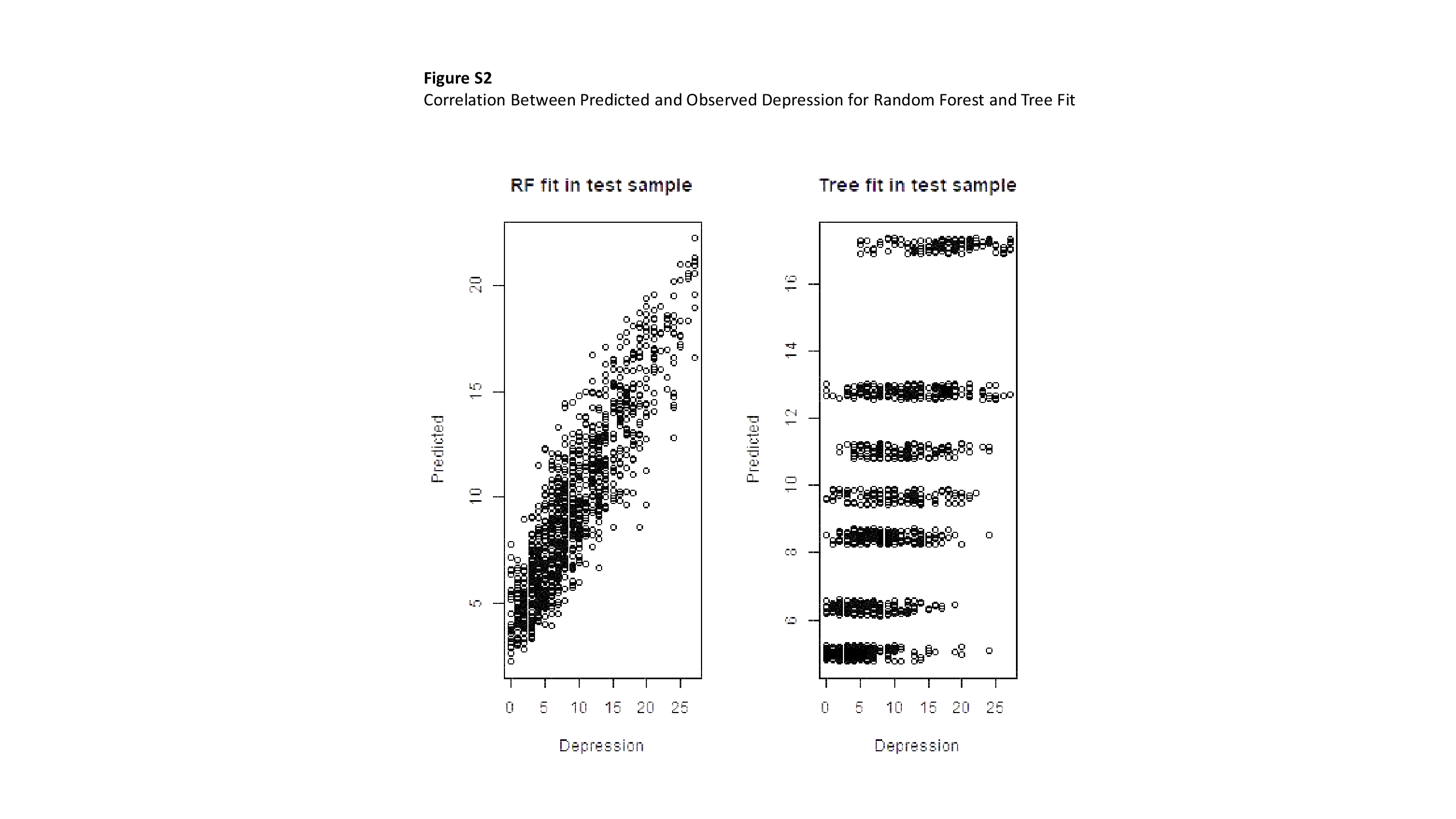

Supplement: Supplementary file 2 [file Image_2.TIFF]

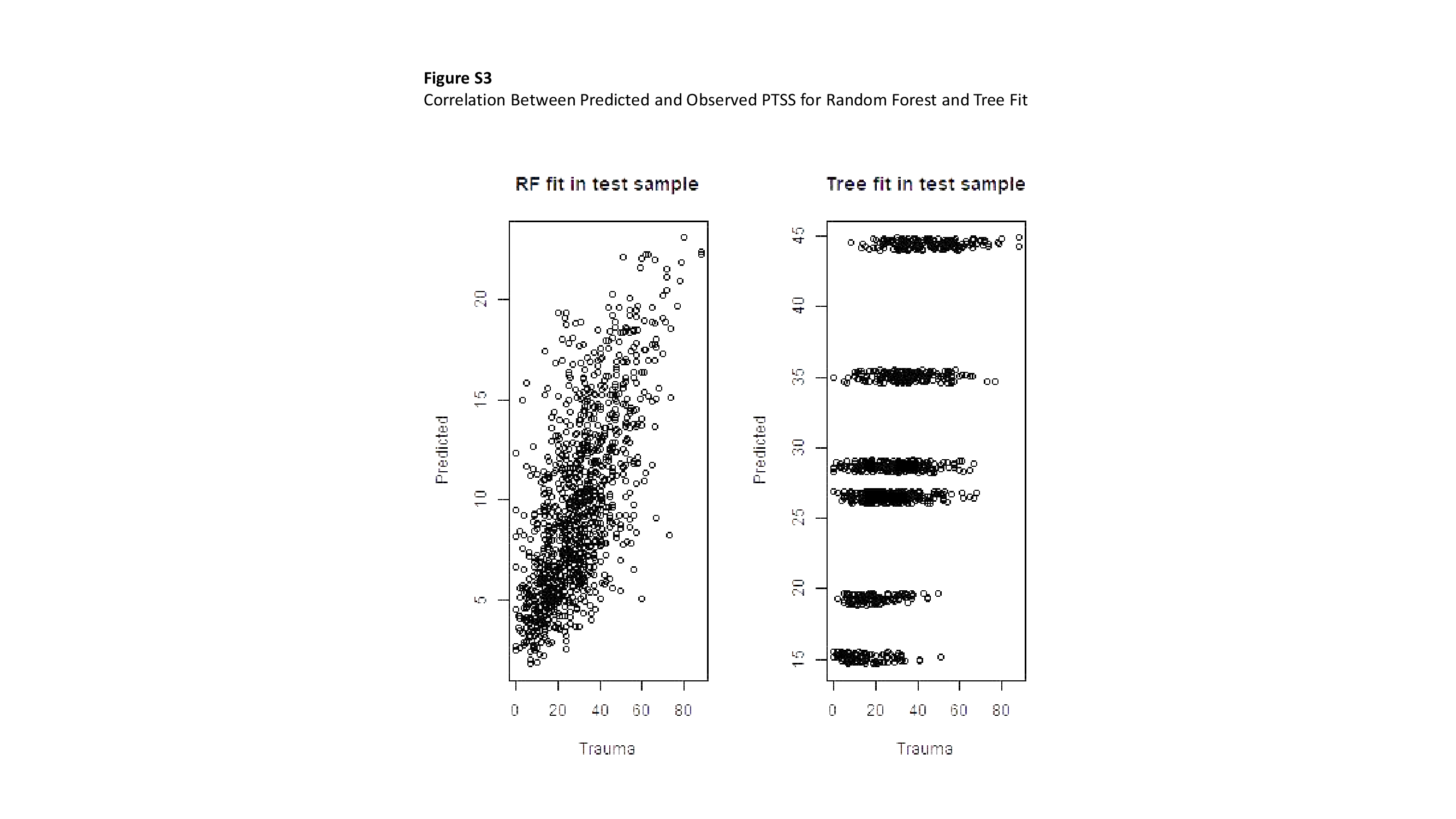

Supplement: Supplementary file 3 [file Image_3.TIFF]
